# Predictors of SLE relapse in pregnancy and post-partum among multi-ethnic patients in Malaysia

**DOI:** 10.1371/journal.pone.0222343

**Published:** 2019-09-20

**Authors:** Syahrul Sazliyana Shaharir, Mohd Shahrir Mohamed Said, Rozita Mohd, Rizna Abdul Cader, Ruslinda Mustafar, Rahana Abdul Rahman

**Affiliations:** 1 Rheumatology Unit, Department of Internal Medicine, Universiti Kebangsaan Malaysia Medical Centre (UKMMC), Jalan Yaacob Latiff, Kuala Lumpur, Malaysia; 2 Nephrology Unit, Department of Internal Medicine, Universiti Kebangsaan Malaysia Medical Centre (UKMMC), Jalan Yaacob Latiff, Kuala Lumpur, Malaysia; 3 Department of Obstetrics and Gynaecology, Universiti Kebangsaan Malaysia Medical Centre (UKMMC), Jalan Yaacob Latiff, Kuala Lumpur, Malaysia; University of Cambridge, UNITED KINGDOM

## Abstract

Flare of Systemic Lupus Erythematosus (SLE) may occur during pregnancy and puerperium. We studied the prevalence and factors associated with SLE relapse during pregnancy and post-partum period in a multi-ethnic SLE cohort. Consecutive SLE patients who attended the outpatient clinic were reviewed for previous history of pregnancies in our institution. Patients who had a complete antenatal, delivery, and post-partum follow up were included. Their medical records were retrospectively analysed to assess the disease activity at pre-pregnancy/conception, during antenatal, and post-partum period. Presence of flare episodes during pregnancy and puerperium were recorded. The pregnancy outcomes recorded include live birth, foetal loss, prematurity and intra-uterine growth restrictions (IUGR). Univariate and multivariable logistic regression with generalized estimating equations (GEE) analyses were performed to determine the factors associated with disease relapse and the pregnancy outcomes. A total of 120 patients with 196 pregnancies were included, with a live birth rate of 78.6%. Four (2.0%) were diagnosed to have SLE during pregnancy. The flare rate in pregnancy was 40.1% while post-partum 17.4%. Majority of the relapse in pregnancy occurred in haematological system (62.3%) followed by renal (53.2%), musculoskeletal (22.1%), and mucocutaneous (14.3%). In GEE analyses, active disease at conception was the independent predictor of SLE relapse during and after pregnancy, whereas older maternal age and Malay ethnicity were associated with higher flare during post-partum. HCQ use was significantly associated with reduced risk of flare in univariate analysis but it was no longer significant in the GEE analyses. Presence of disease flare in pregnancy was significantly associated with prematurity. In conclusion, pregnancy in SLE need to be planned during quiescent state as pre-pregnant active disease was associated with disease relapse in both during and after pregnancy. Malay patients had an increased risk of post-partum flare but further larger prospective studies are needed to confirm the association between pregnancies in the different ancestral background.

## Introduction

Systemic lupus erythematosus (SLE) is an autoimmune disease which commonly affects young women in their reproductive age [1]. It is characterized by production of autoantibodies resulting in inflammation of multiple organs with a relapse remitting pattern [2]. SLE is a heterogenous disease with a very wide spectrum of disease manifestations across different regions and ethnicities [3]. Since SLE predominantly affects young women, pregnancy is not rare as fertility is maintained among majority of the patients [4]. Despite favourable outcome of pregnancy among SLE patients [5], it continues to pose a significant challenge as the effect of pregnancy on SLE activity are still debatable in the literature. This is because the nature of SLE itself is a relapse-remitting disease and most of the studies had lack of controls for direct comparisons.

Some studies have reported an increase risk of flare relapse during pregnancy [6–10], whereas others reported no increased risk [11, 12]. Despite high relapse risk, earlier studies reported that the flares were generally mild such as arthritis, fatigue, and cutaneous manifestations [10, 13–15] and comparable to non-pregnant flares [7, 8, 10, 11, 13, 16]. On the other hand, studies on the risk of lupus flare during postpartum revealed conflicting results [17]. The LUMINA groups demonstrated decreased disease activity after pregnancy [18]. However, the discrepancy of the flare incidences varied due to different definitions of flare and active disease used. Most studies were published before the validation of the modification of disease activity indices in pregnancy such as Systemic Lupus Erythematosus-Pregnancy Disease Activity Index (SLEPDAI), the Lupus Activity Index in Pregnancy (LAI-P), and the modified Systemic Lupus Activity Measure (m-SLAM-P) [19, 20].

Managing lupus pregnancies are challenging as most of the immunosuppressive medications are not safe in pregnancy [21]. Therefore, characteristics of patients who are at high risk of relapse need to be identified so that appropriate pregnancy plan can be implemented. At present, there is very limited data on pregnancy outcome among women with SLE with a higher prevalence of renal involvement. Published studies are mainly derived from the Caucasian populations which have less severe disease; less than a third of them had major organ or renal manifestations [9, 13, 22]. SLE patients in Asia including Malaysia have a higher rate of renal involvement of up to two-thirds of their SLE cohorts [23, 24]. Much of the pregnancy data on Asian patients to date has come from Chinese ethnics [25–30] and, to a lesser extent, Korean [31] patients. Malaysia is a multi-ethnic country with Malay, Chinese, and Indian constituting the majority of the population, and the influence of different ancestries in the SLE course during pregnancy or post-natal period is not well-studied. It is very imperative to delineate the risk of flare in SLE patients so that appropriate pre-pregnancy counselling and antenatal care can be personalized.

## Methodology

### Patients

This was a cross-sectional study conducted from January 2016 until December 2018 on consecutive SLE patients who attended the outpatient Rheumatology and Nephrology Clinic in Universiti Kebangsaan Malaysia Medical Center (UKMMC). Patients who had history of pregnancy/pregnancies with a regular antenatal and post-partum care at our institution were identified and included. All patients fulfilled the American College of Rheumatology Classification Criteria (ACR) 1997 [32] or Systemic Lupus International Collaborating Clinics Classification Criteria (SLICC) for SLE 2012 [33]. Each pregnancy was counted as a separate observation. Patients with inadequate data or had antenatal follow-up in other hospitals were excluded. All patients have given informed consent and this study has obtained approval from the Universiti Kebangsaan Malaysia Medical Centre (UKMMC) ethics committee (FF-2016-297).

Their medical records were analysed and baseline maternal information that were recorded include: 1) socio-demographic data (age at pregnancy, race/ethnic), 2) past obstetric history, 3) SLE disease characteristics such as disease onset, system manifestations, and immunological features, and 4) co-morbidities such as hypertension, diabetes mellitus, and thyroid disease. Lupus nephritis (LN) was defined according to the American College of Rheumatology (ACR) criteria by persistent proteinuria > 0.5 g/24h, or the presence of cellular casts, persistent hematuria, or renal biopsy results consistent with LN [34]. Antiphospholipid syndrome (APLS) was defined as positive aPL, combined with a history of thrombosis, foetal loss at ≥ 10 weeks gestation, or 3 prior first-trimester miscarriages [35]. Immunologic characteristics of the patients were recorded and these included positive antiphospholipid antibodies (aPL) i.e. anticardiolipin (aCL) IgG or IgM and/or lupus anticoagulant (LA), and extractable nuclear antigen (ENA) including anti-Ro and La.

### Assessment of disease activity

Disease activity at conception and pregnancy was determined retrospectively by using the SLE-Pregnancy Disease Activity Index (SLEPDAI) [20]. SLEPDAI has been modified to assess lupus disease activity in pregnancy in an effort to help differentiate pregnancy complications from lupus flare. Disease remission was defined as SLEPDAI score of 0 in the clinical items [36, 37] with prednisolone dose of ≤ 10 mg daily. Complete lupus nephritis remission was defined by proteinuria of < 0.03 g/day [38]. Presence of disease relapse or flare-up was defined as new onset or worsening of disease in a previously normal or affected organ/system [39]. Meanwhile, for patients who were already had an active disease at conception but experienced no worsening of activity through-out the pregnancies, they were categorized as “persistent stable active disease” group.

### Immunosuppressants and Hydroxychloroquine (HCQ) treatment

Information on the hydroxychloroquine (HCQ) and immunosuppressive medications particularly azathioprine and ciclosporine A at conception, during pregnancy, and post-partum were retrieved from the electronic prescription and medical records. Since the impact of cessation or stopping HCQ on disease activity in pregnancy may take up to 2–3 months [40], the exposure to HCQ among our subjects were divided into 2 groups:

1. Group 1: No exposure to HCQ treatment in the 3 months prior to pregnancy or stopped taking HCQ at any trimester of the pregnancy, and
2. Group 2: Took HCQ 3 months prior and throughout the pregnancy.

Post-partum HCQ use was defined as continuous use up to 3 months post partum [41].

The use of other immunosuppressive medications 3 months prior and during pregnancy were also recorded.

### Pregnancy outcomes

In this study, two pregnancy outcomes were assessed which are: i) live birth: defined as pregnancy which resulted in the birth of a living child, and ii) foetal loss: defined as all pregnancies that did not end with a live birth, including spontaneous abortions, therapeutic abortions, stillbirths, or intrauterine foetal deaths [42]. Presence of foetal complications ie premature births and intrauterine growth restrictions (IUGR) were also recorded.

### Statistical analyses

Quantitative variables were reported as mean and SD, or median and range, depending on the distribution. Absolute and relative frequencies were used for categorical variables. The impact of clinical and laboratory characteristics on the disease relapse in pregnancy and post-partum were tested by univariate analysis using χ ^2^ or Fisher’s exact tests (if one or more of the variable cells had an expected frequency of five or less) for categorical variables. Meanwhile, for continuous variables, independent student T-tests or Mann-Whitney tests were performed, depending on the distribution of the data. For comparisons of continuous variable between three or more groups, one-way analysis of variance (ANOVA) was used to for normally distributed variables while Kruskal-Wallis was used for non-normally distributed variables. Binary logistic regression analyses were conducted using generalized estimating equations (GEE) to adjust for multiple pregnancies and possibility of disease relapses that may be correlated within a patient. All variables that were significant in univariate analyses with p<0.05 were included as independent variables in the model, in order to determine the predictors associated with relapse of SLE in pregnancy and post-partum period. Analyses were performed using the SPSS version 18.0 (SPSS Inc. Chicago IL, USA) package.

## Results

A total of 120 patients with 196 pregnancies were analysed. Almost two-third of the pregnancies occurred after the year of 2010 (n = 125, 63.8%) while the rest occurred from 1998–2009 (n = 71, 36.2%). Majority of the SLE patients in this study were Malay (66.7%, n = 80) followed by Chinese (27.5%, n = 33), Indian (5.0%, n = 6), and 1 Arab (0.8%). The mean age at conception was 30.9 ± 4.1 years while the average disease duration was 7.4 ± 5.1 years. Majority of the patients had musculoskeletal involvement (n = 89, 74.2%) and more than half of the patients had lupus nephritis (n = 66, 55%). Lupus nephritis (LN) tend to be more prevalent among Chinese as compared to other ethnics, with p = 0.06. Of the 66 patients with lupus nephritis (LN), almost half of them had class IV with/without V (n = 29, 43.9%). Antiphospholipid syndrome (APLS) tend to occur among patients with Indian and Arab ethnic group (p = 0.03) and they significantly had a higher history of recurrent miscarriages ≥1 (p = 0.04).

Anti-cardiolipin IgG and IgM status were available in 118 patients while lupus anticoagulant status was available in 107 patients. Meanwhile, anti-Ro/La, anti-Sm and anti-RNP status were available in 105 patients. There were no significant differences between these auto-antibody statuses among different ethnicities. Table 1 illustrates the baseline characteristics of all pregnant SLE patients and according to the ethnicities.

**Table 1:** Baseline characteristics of all pregnant SLE patients and according to the ethnicities.

Parameters	All patients, n = 120 Frequency (%)/ Mean ± S.D	Malay (n = 80, 66.7%)	Chinese, (n = 33, 27.5%)	Others* (n = 7, 5.8%)	*p*
Age at conception (years)	30.9 ± 4.1	30.6 ± 3.8	31.5 ± 4.5	30.5 ± 4.8	0.54
Age of SLE diagnosis (years)	24.3 ± 5.6	24.1 ± 4.9	24.2 ± 5.8	23.3 ± 5.7	0.78
SLE duration at conception (years)	7.4 ± 5.1	7.1 ± 4.9	7.9 ± 5.7	8.6 ± 4.6	0.42
System manifestation, n (%)					
Musculoskeletal	89 (74.2)	63 (78.8)	20 (60.6)	6 (85.7)	0.06^&^
Haematological	77 (64.2)	55 (68.8)	19 (57.6)	3 (42.9)	0.10
Lupus Nephritis	66 (55.0)	39 (48.8)	22 (66.7)	5 (71.4)	0.06^#^
Muco-cutaneous	64 (53.3)	45 (56.3)	15 (45.4)	4 (57.1)	0.45
Neuropsychiatric	11 (9.2)	7 (8.8)	2 (6.1)	0 (0)	0.71
APLS	24 (12.2)	9 (11.3)	0 (0)	2 (28.6)	0.03^&^
Renal biopsy, n = 66					
No biopsy, n (%)	19 (28.8)	10 (15.2)	7 (10.6)	2 (9.1)	0.55
Class II, n (%)	1 (1.5)	1 (1.5)	0 (0)	0 (0)	
Class III (± class V), n (%)	12 (18.2)	8 (12.1)	3 (4.5)	1 (1.5)	
Class IV (± class V), n (%)	29 (43.9)	19 (28.8)	8 (12.1)	2 (3.0)	
Class V, n (%)	5 (7.6)	1 (1.5)	4 (6.1)	0 (0)	
Anti-dsDNA positive, n (%)	98 (84.5)	65 (81.2)	28 (84.8)	5 (71.4)	0.61
aPL status^¶^, n (%)					
LA positive	25 (23.4)	14 (17.5)	8 (24.2)	3 (42.9)	0.42
aCL IgG positive	30 (25.4)	20 (25.0)	7 (21.2)	3 (42.9)	0.43
aCL IgM positive	28 (23.7)	20 (25.0)	8 (24.2)	0 (0)	0.65
ENAs status^¥^, n (%)					
Anti Ro/La positive	30 (28.6)	21 (26.3)	8 (24.2)	1 (16.7)	0.99
Anti-Sm positive	27 (25.7)	15 (18.8)	9 (27.3)	3 (50.0)	0.08
Anti-RNP positive	24 (23.1)	16 (20.0)	5 (15.2)	3 (50.0)	0.23
Obstetric history, n = 192					
Primigravida, n (%)	74 (37.8)	47 (35.6)	23 (50.0)	4 (22.2)	0.06^&^
Prior foetal loss, n (%)	26 (13.3)	17 (12.9)	4 (8.7)	5 (27.8)	0.07^$^
History of recurrent miscarriages ≥ 2, n (%)	16 (8.2)	9 (6.9)	3 (6.5)	4 (22.2)	0.04^$^
Disease activity at conception (n = 192)					
Active disease	61 (31.8)	42 (32.1)	12 (27.9)	7 (38.9)	0.48
Active haematology	22 (11.5)	14 (10.7)	6 (14.0)	0 (0)	0.17
Active LN	26 (19.8)	26 (19.8)	5 (11.6)	2 (11.1)	0.36
Active musculoskeletal	7 (3.6)	6 (4.6)	1 (2.3)	0 (0)	0.56
Active muco-cutaneous	9 (4.7)	5 (3.8)	1 (2.3)	3 (16.7)	0.04^$^
Active pulmonary	1 (0.5)	1 (0.8)	0 (0)	0 (0)	1.00

aCL = anticardiolipin, APLS = antiphospholipid syndrome, ENAs = Extractable nuclear antigens, LA = lupus anticoagulant, LN = lupus nephritis

*Others: 6 Indians and 1 Arab

^&^Chinese compared to Malays and Indians

^#^Malays compared to Chinese and Indians

^$^Others compared to Malay and Chinese

¶ Total patients with aCL = 118, LA = 107

^¥^ Total patients with ENAs (anti-Ro/La, anti-Sm and anti-RNP) = 105.

### Disease activity and pattern of disease relapse in pregnancy

From the total of 196 pregnancies, four (2.0%) were newly diagnosed with SLE during pregnancy. In 192 pregnancies, 71 (37%) were unplanned pregnancy and 61 (31.8%) were in active disease at conception with median SLEDAI score of 6 (IQR 5). The commonest active system/organ at conception was lupus nephritis (n = 33, 17.2%), followed by haematological (n = 22, 11.5%), mucocutaneous (n = 9, 4.7%), and musculoskeletal (n = 7, 3.6%). One patient (0.5%) had active interstitial lung disease or pneumonitis. The patient with active lung pneumonitis was advised against pregnancy, and refused termination of pregnancy. There was no significant difference between active disease at conception with ethnicity. However, Indian patients were noted to have more active mucocutaneous lupus at conception compared to other ethnicities (p = 0.04), as illustrated in Table 1.

From the 192 pregnancies of patients with pre-existing SLE, 77 of them (40.1%) had a flare or worsening disease activity while 16 (8.3%) had persistent stable active disease from conception throughout the pregnancy. Majority of the relapse occurred in haematological system (n = 48, 62.3%) followed by LN (n = 41, 53.2%), musculoskeletal (n = 17, 22.1%), mucocutaneous (n = 11, 14.3%), cardiorespiratory (n = 3, 3.9%), ophthalmology (n = 1, 1.3%), and gastroenterology (n = 1, 1.3%). Majority had disease relapse in the first trimester (n = 34, 44.2%) followed by second (n = 26, 33.8%) and third trimester (n = 17, 22.1%). The average timing of relapse was at 17.6 ± 9.6 weeks of gestation.

Meanwhile, from the 16 patients who had persistent stable active disease, majority of them had renal (n = 9, 56.3%) followed by haematology (n = 5, 31.3%), skin (n = 1, 6.3%) and pulmonary (n = 1, 6.3%).

In pregnancy, a total of 65 (33.2%) patients were on azathioprine, 21 (10.7%) were on cyclosporine A while 23 (11.7%) were on combination of azathioprine and Ciclosporine. Only 69 patients (35.2%) received a continuous hydroxychloroquine (HCQ) 3 months prior to conception and throughout the pregnancy while 95 patients (48.5%) had HCQ during post-partum period.

### Predictors of relapse in pregnancy

A total of 49.4% of pregnancies with active disease at conception had relapse or worsening of disease activity during the course of pregnancy. In contrast, only 20.0% of those who were in remission at conception experienced relapse of disease during pregnancy (p<0.001). Disease relapse in pregnancy was significantly lower among Malays (58.4% vs 74.8%, p<0.05) as compared to other ethnics while Chinese patients had higher tendency to have relapse in pregnancy as compared to other ethnics (31.2% vs 16.5%, p = 0.05). Higher rate of relapse occurred among those with shorter median duration of pre-pregnancy remission of 1 (IQR 12) month as compared to 12 (IQR 30) months among those who had no relapse, p<0.001.

SLE patients with history of musculoskeletal involvement had a lower rate of disease relapse in pregnancy (67.5% vs 82.6%, p<0.05). In contrast, patients with active haematological SLE during pre-pregnancy experienced a significant worsening of disease activity during pregnancy (p<0.05). Patients who had a continuous hydroxychloroquine treatment since at least 3 months’ pre-pregnancy and throughout pregnancy tend to have lower prevalence of disease relapse (28.6% vs 40.9%, p = 0.05). Table 2 illustrates the factors associated with disease relapse in pregnancy among the 192 pregnancies.

**Table 2:** The associated factors of disease relapse in pregnancy among SLE women.

Variables	No Relapse (n = 115)	Relapse (n = 77)	p
Age at conception (years)	31.1 ± 4.1	30.6 ± 4.0	0.69
Disease duration at conception (years)	7.7 ± 5.3	7.3 ± 4.7	0.39
**Ethnic, % (n)**			
Malay	74.8 (86)	58.4 (45)	0.04*
Chinese	16.5 (19)	31.2 (24)	0.05^**#**^
Indian	7.0 (8)	7.8 (6)	0.59
Others	1.7 (2)	2.6 (2)	0.89
**Prior SLE system involvement**			
Lupus nephritis, % (n)	59.1 (68)	53.2 (41)	0.46
Haematology, % (n)	61.7 (71)	74.0 (57)	0.09
Musculoskeletal, % (n)	82.6 (95)	67.5 (52)	0.02
Mucocutaneous, % (n)	55.7 (64)	50.6 (39)	0.57
Neuropsychiatry, % (n)	7.5 (9)	5.2 (4)	0.57
APLS, % (n)	10.4 (12)	14.3 (11)	0.49
Pregnancy prior to 2009, % (n)	33.9 (39)	39.0 (30)	0.54
Duration of remission (months)	12 (IQR 30)	1 (IQR 12)	<0.001
Active disease at conception, % (n)	20.0 (23)	49.4 (38)	<0.001
Active system at conception			
Lupus nephritis, % (n)	13.0 (15)	23.4 (18)	0.12
Haematology, % (n)	5.2 (6)	20.8 (16)	0.002
Musculoskeletal, % (n)	2.6 (3)	5.2 (4)	0.44
Muco-cutaneous, % (n)	3.5 (4)	6.5 (5)	0.49
Low C3 or C4 pre-pregnancy	43.5 (50)	48.1 (37)	0.58
Anti-dsDNA positive pre-pregnancy, % (n)	45.2 (52)	57.1 (44)	0.14
Group 2 HCQ use**, % (n)	40.9 (47)	28.6 (22)	0.06

APLS = antiphospholipid syndrome, HCQ = Hydroxychloroquine, NPSLE = neuropsychiatric lupus

*Malay vs non-Malays

^#^Chinese vs non-Chinese

** took HCQ 3 months prior and throughout the pregnancy

### Post-partum relapse and the associated factors

Patient with active pneumonitis passed away at 19 weeks POA due to severe respiratory failure. From a total of 195 pregnancies, 34 (17.4%) of them had a flare or worsening of disease activity during post partum period. The median time of relapse occurred at 10 (IQR 13) weeks post-partum. Majority had relapse LN (n = 17), followed by hematological (n = 14 patients), musculoskeletal (n = 3), cutaneous (n = 1) and serositis (n = 1).

Factors that were significantly associated with relapse of disease during post-partum period include younger age at conception, Malay ethnic, shorter duration of pre-pregnant remission, active disease at conception and pre-pregnancy positive anti-dsDNA antibody (all p<0.05). Patients who received hydroxychloroquine in pregnancy and post-partum period had lower rate of relapse (p<0.05). Table 3 illustrates the clinical and socio-demographic factors associated with SLE relapse in post-partum in 195 pregnancies.

**Table 3:** The associated factors of disease relapse in post-partum in 195 pregnancies of women with SLE.

Variables	No Relapse (n = 161)	Relapse (n = 34)	p
Age at conception (years)	31.3 ± 3.9	29.1 ± 3.9	0.004
Disease duration at conception (years)	7 (IQR 7)	6 (IQR 6.3)	0.31
Malay, % (n)	63.4 (102)	85.3 (29)	0.02*
Chinese, % (n)	26.1 (42)	11.8 (4)	0.08^#^
Indian, % (n)	8.1 (13)	2.9 (1)	0.26
Pregnancy prior to 2009, % (n)	36.6 (59)	35.3 (12)	1.00
Duration of remission (months)	10 (IQR 24)	3.5 (IQR 18)	0.03
Active disease at conception, % (n)	28.0 (44)	47.1 (16)	0.04
Active disease in pregnancy	28.0 (44)^$^	47.1 (16)	0.04
Anti-dsDNA positive pre-pregnancy, % (n)	46.5 (73)^$^	67.6 (23)	0.04
Low C3 or C4 pre-pregnancy	43.9 (69)^$^	50.0 (17)	0.58
Anti-dsDNA positive in pregnancy	46.5 (73)	48.5 (16)	0.56
Low C3 or C4 in pregnancy	49.7 (80)	41.2 (14)	0.45
Group 2 HCQ**, % (n)	38.5 (62)	17.6 (6)	0.03
HCQ in post-partum	52.2 (84)	32.4 (11)	0.04
Prior SLE system involvement,			
Lupus nephritis, % (n)	57.1 (92)	58.8 (20)	1.00
Haematology, % (n)	64.6 (104)	73.5 (25)	0.42
Musculoskeletal, % (n)	74.5 (120)	76.5 (26)	1.00
Mucocutaneous, % (n)	51.6 (83)	58.8 (20)	0.45
NPSLE, % (n)	8.1 (13)	0 (0)	0.13
APLS, % (n)	12.4 (20)	11.8 (4)	1.00
At conception active disease			
Lupus nephritis, % (n)	15.3 (24)	26.5 (9)	0.14
Haematology % (n)	9.6 (15)	20.6 (7)	0.08
Musculoskeletal, % (n)	3.2 (5)	5.9 (2)	0.61
Muco-cutaneous, % (n)	3.8 (6)	8.8 (3)	0.20
Pregnant active system			
Lupus nephritis, % (n)	24.2 (39)	35.3 (12)	0.20
Haematology. % (n)	21.7 (35)	35.3 (12)	0.12
Musculoskeletal, % (n)	8.7 (14)	5.9 (2)	0.74
Mucocutaneous, % (n)	6.2 (10)	8.8 (3)	0.70

APLS = antiphospholipid syndrome, HCQ = Hydroxychloroquine, NPSLE = neuropsychiatric lupus

*Malay vs other ethnics

^#^Chinese vs other ethnics

^$^In 157 pregnancies as 4 SLE patients were diagnosed in pregnancy

** took HCQ 3 months prior and throughout the pregnancy

### Logistic regression analysis with generalized estimating equations (GEE)

Results of the generalized estimating equations analysis (GEE) revealed that the independent predictors of disease relapse in pregnancy was active disease at conception. Meanwhile, the independent predictors of disease relapse post-partum were active disease in pregnancy, advance age at conception and Malay ethnicity (Table 4).

**Table 4:** Logistic regression with generalized estimating equations analyses (GEE) of predictors of disease relapse in pregnancy and post-partum period among SLE patients.

Clinical variables	B Coefficient	OR (95% C.I)	*P*
Relapse in pregnancy			
Active disease at conception	1.54	4.66 (1.03–21.38)	0.04
Chinese	0.61	1.84 (0.63–5.37)	0.27
Musculoskeletal	-0.66	0.82 (0.22–1.29)	0.14
Malay	-0.51	0.60 (0.25–1.47)	0.27
Active LN at conception	-0.43	0.65 (0.15–2.69)	0.55
Active haematology at conception	-0.26	0.77 (0.17–3.51)	0.73
Duration of remission	0.02	1.11 (0.99–1.04)	0.17
Hydroxychloroquine in pregnancy	-0.54	0.58 (0.31–1.11)	0.10
Relapse post-partum			
Age at conception	0.13	1.14 (1.04–1.26)	0.01
Active disease at conception	0.91	2.48 (1.08–5.69)	0.03
Malay	1.35	3.87 (1.29–11.51)	0.02
HCQ in pregnancy	-0.72	0.49 (0.13–1.82)	0.28
HCQ post partum	-0.54	0.59 (0.19–1.83)	0.35
Duration of remission	0.01	1.01 (0.98–1.05)	0.66

HCQ = hydroxychloroquin

### Pregnancy outcomes and their associations with SLE disease activity

Live-birth rate in this cohort was 78.6% (n = 54) while 21.4% (n = 42) had foetal loss. Pregnancies with foetal loss was significantly associated with active disease at conception as 56.1% (n = 23) of them conceived when the disease was active. Patients with active LN at conception had a higher rate of foetal loss (31.7% vs 13.2%, p = 0.009). In addition to that, significantly more pregnancies with active disease and LN had spontaneous early miscarriage at less than 12 weeks POA (33.3% vs 15.1%, p = 0.02). In contrast, majority of successful pregnancies with live-birth (74.8%, n = 113) had quiescent disease at conception (p<0.001). There were no associations between foetal loss with relapse of disease or active disease during pregnancy.

Prematurity occurred in 56 (31.6%) while 38 (26%) had intra-uterine growth restrictions (IUGR). Patients who experienced flare of SLE during pregnancies had a higher rate of premature births (52.1% vs 17.3%, p<0.001) but no significant associations with IUGR (33.9% vs 20.2%, p = 0.09).

## Discussion

Despite the controversial and inconsistent data on disease activity of SLE during pregnancy, majority of the researchers agree that the pregnancy hormonal changes which induce Th-2 response contribute to an increase in flare risk [43, 44]. In our cohort, the rate of relapse was approximately 40% and this was lower than those reported in the earlier reported rate of 40–75% in the literatures published before the year 2000 [6–8, 10]. The rate of flare in pregnancy continued to decline in the year 2000–2010, ranging from 13–40% [9, 13, 40, 45, 46], and majority of the reports from 2010 onwards demonstrated lower flare episodes of less than 30% [28, 47, 48]. The reported post-partum relapses were even lower, ranging from 4.9–20% [13, 49–51]. The improvement in the flare frequency over the years may reflect a more tactful management of pregnancy among SLE patients. However, different methodology, composition of patients, and definition of flare may also be the actual causes that have contributed to such discrepancies observed in the literature.

In our study, patients having active disease 6 months prior to or at conception carried a significant 4-fold increased risk of disease flare in pregnancy and a 2-fold increased relapse during post-partum. This finding corroborates with many other previous studies [9, 30, 31, 52, 53]. In patients with quiescent disease prior to pregnancy, the flare rate is estimated at 5–30% but it can go up to 75% among patients with active disease [50, 54]. Despite a more robust association between LN and disease relapse in pregnancy [7, 9, 25, 31, 46, 48, 55–57], our study did not demonstrate any significant association between them. Two studies with predominant Caucasian patients also observed no significant increase risk of disease activity among their LN patients as compared to non-pregnant controls [58], and the incidence of renal flare during pregnancy and 6 months after delivery was similar to that observed before pregnancy [45]. However, in our study, LN patients with active disease at conception had significantly higher rate of early trimester miscarriages. Therefore, any worsening of disease activity in the later part of pregnancy was not able to be captured. Further sub-analyses excluding patients with miscarriages in our study revealed that patients with active LN at conception were indeed significantly associated with an increase in flare episodes.

Apart from renal flare, majority of the relapse episodes among our patients occurred in haematological system and this observation is consistent with the Chinese cohort studies [26, 30] and the Hopkins Lupus Cohort [59]. In contrast, patients with history of musculoskeletal lupus had lower rate of relapse and this was in contrast with many other earlier Caucasian or European cohorts [10, 13, 60]. We also observed that higher maternal age was independently associated with relapse of disease in post-partum period. This observation has not been reported in the literature.

A continuous hydroxychloroquine (HCQ) use 3 months prior to and during pregnancy was associated with reduced flare episodes in pregnancy and post-partum period. However, the role of HCQ use in preventing relapse was less significant in our study as it was no longer a significant factor in the GEE analyses. This might be due to the fact that HCQ did not prevent more severe complications of proteinuria or thrombocytopenia in pregnancy [40]. Nevertheless, our study further reinforces the importance of continuing HCQ treatment during pregnancy in reducing the risk of flare during and after pregnancy [40, 61].

Interestingly our study found that Chinese patients had a higher rate of flares during pregnancy while Malay patients had an increase risk of relapse during post-partum period. This observation can be explained by the fact that Chinese patients tend to have prior renal involvement and the literature reported a higher risk of relapse in patients with prior history of LN [7, 9, 11, 48, 62]. There are considerable differences in the post-natal practices among different ethnics in Malaysia [63]. Medicinal plant or herbs intake is a common practice during post-natal period among Malay [64] and whether this may influence the SLE disease activity is not well studied. Other postulation include possible non-adherence to medications due to fear of harming their breastfed infants. However, all these postulations need to be confirmed in future prospective studies.

Little is known whether disease activity and relapse pattern of SLE in pregnancy vary across different ethnics and regions, as there are no large head-to-head studies to date. In a cohort of Chinese SLE patients with well-planned pregnancies i.e. quiescent disease of at least 6 months prior to conception, their relapse rate was considerably high at 21.4% which mainly affected the renal [25]. On the other hand, a recent study with predominantly Caucasian patients revealed that their relapse rate was lower at 17%, despite their cohort of patients having mild to moderate active disease at baseline of pregnancy and higher prevalence of LN [47]. In addition to that, the incidence of worsening of proteinuria of above 500 mg/day from baseline was low at 2.8% in their study [47]. In contrast, disease activity in pregnancy was higher among the African-American women in a multi-ethnic US study [9]. Indeed, among general non-pregnant SLE populations, African-American and Asian ethnics exhibit more severe lupus manifestations involving the renal [65]. However, there were many possible confounding factors that need to be identified. Further prospective head-to-head studies are needed to address the influence of different ancestries on disease activity during pregnancy. Table 5 illustrates the difference in the rate and pattern of SLE relapse across different geographical regions of patients.

**Table 5:** The prevalence and characteristics of SLE flares in pregnancy across different ethnics and geographical regions.

Study	Europe/UK	US/ Canada	South American	Asia	Middle East
Flare rate in pregnancy	13.5–74% [8, 10, 12, 13, 15, 22, 67]	17.7%-68% [7, 40, 68–70]	37.9–85.3% [55, 71]	13–50.2% [26, 48, 50, 56, 72–76]	20.2–56.5% [51, 57, 77]
No difference with controls	[11]	[52, 78, 79]			
Increase flare vs control	[8]	[7, 80]		[6]	
Organ/system relapse					
MSK	[10, 13, 67]	[68]	[55]	[50]	[57]
Haematology	[10]			[26, 30, 50]	[51]
Renal		[7]	[55, 71]	[26, 30]	[51, 57, 77]
Skin	[13, 67]	[68]	[71]	[50]	[57, 77]
Constitutional	[13]	[7, 68]			
Predictor					
Renal	[81]		[55]	[48, 56, 76]	[57]
Pre-pregnant Low C3/C4	[81]			[30]	
Pre-pregnant Anti-dsDNA				[30]	[77]
Active disease at conception		[22, 52]	[55]	[30, 48, 74]	
African- American ancestry		[9]			
Primigravida			[55]		
Non-HCQ		[40, 70]			

MSK = musculoskeletal, HCQ = hydroxychloroquine

There were several limitations in our study and this includes the retrospective nature of this study. The assessment of lupus activity during pregnancy can be difficult as physiological changes like alopecia, palmar erythema, chloasma, and increase in proteinuria due to increased glomerular filtration rate, may be mistakenly thought as flare of the disease. Another particular challenge in pregnant patients with SLE is differentiating preeclampsia from active lupus nephritis as both may co-exist [66]. Patients with a significant proteinuria due to the sequelae of previous LN may also experience up to double of protein loss in the absence of active nephritis due to increased renal blood flow in pregnancy [44]. As majority of our active LN patients did not have renal biopsies to confirm the cause of worsening proteinuria, the diagnoses of flare episodes were highly dependent on the physicians’ judgement. We also included only those patients with a complete data from pre-pregnancy until 3 months post-partum and this may contribute to potential bias as patients who defaulted post-partum follow up may have lower rates of flare during pregnancy. Nonetheless, with the limitations, we have identified several possible risk factors associated with relapse during pregnancy and post-partum period.

## Conclusions

Active disease at conception was independently associated with increased risk of relapse in pregnancy and post-partum period. In addition to that, Malay patients and conception that occurred in older maternal age had an increased risk of relapse during post-partum period. Further larger prospective studies are needed to further confirm this observation.

## Supporting information

S1 Appendix Raw data. (SAV)
